# LINC01296 promotes proliferation of cutaneous malignant melanoma by regulating miR-324-3p/MAPK1 axis

**DOI:** 10.18632/aging.204413

**Published:** 2022-12-02

**Authors:** Kang Wang, Qing Luo, Yingfeng Zhang, Xin Xie, Wenhao Cheng, Qiunan Yao, Yingying Chen, Hong Ren, Jiuping Li, Zuanqin Pan

**Affiliations:** 1 Department of General Surgery, Gaoyou People’s Hospital, Yangzhou 225600, China; 2 Department of Dermatology, The First People’s Hospital of Lianyungang, Lianyungang Clinical Medical College of Nanjing Medical University, Lianyungang 222002, China; 3 Department of General Medicine, The First People’s Hospital of Lianyungang, Lianyungang Clinical Medical College of Nanjing Medical University, Lianyungang 222002, China

**Keywords:** cutaneous malignant melanoma, LINC01296, miR-324-3p, MAPK1, proliferation

## Abstract

Objective: To investigate the functions and potential molecular mechanism of LINC01296 regarding the progression of cutaneous malignant melanoma (CMM) by the regulation of miR-324-3p/MAPK1 axis.

Methods: The candidate differential lncRNAs of CMM were selected from GEPIA database, and quantitative real-time PCR (qRT-PCR) was utilized to assess the expression level of LINC01296 in human CMM tissues and cell lines. Cell proliferation assay, Colony formation assay, Ethynyl-2’-deoxyuridine (EDU) assay *in vitro* and tumorigenicity assays in nude mice *in vivo* were performed to examine the functions of LINC01296. Bioinformatics analysis, luciferase reporter assay and rescue experiments were also gained an insight into the underlying mechanisms of LINC01296 in CMM cell lines by miR-324-3p/MAPK1 axis.

Results: In this study, the up-regulation of LINC01296 was found in CMM tissues and cell lines. Functionally, the over-expression of LINC01296 promoted the proliferation in CMM cell lines. In addition, immunochemistry analysis confirmed that the levels of MAPK1 and Ki-67 in sh-LINC01296-xenografted tumors was weaker than that in sh-NC-xenografted tumors. Then, bioinformatics analysis confirmed that LINC01296 interacted with miR-324-3p. Further investigations showed that MAPK1, which collected from the potential related genes of LINC01296, was the conjugated mRNA of miR-324-3p by luciferase reporter assay. Finally, the rescue experiments suggested the positive regulatory association among LINC01296 and MAPK1, which showed that MAPK1 could reverse the promoting-effect of LINC01296 in CMM cells *in *vitro**.

Conclusions: Therefore, our findings provided insight into the mechanisms of LINC01296 via miR-324-3p/MAPK1 axis in CMM, and revealed an alternative target for the diagnosis and treatment of CMM.

## INTRODUCTION

Globally, cutaneous malignant melanoma (CMM), as one of the fastest growing and the most common surface malignancies, is derived from melanocytes and frequently occurs in the areas where are rarely exposed to ultraviolet light [1, 2]. Once CMM enters the rapid growth phase, the prognosis of CMM patients is extremely poor with high mortality [3, 4]. The National Cancer Institute predicts that by 2020 the United States will have approximately 100,000 new cases of melanoma and 6,850 deaths [4, 5]. At present, there is much room for improvement in the treatment of CMM, due to the insensitive to radiotherapy and chemotherapy [6]. Thus, early surgical excision is the optimal treatment option for CMM and is recognized as the most significant and promising therapy [7]. CMM is mostly progressed from sputum or pigmented plaque, and the underlying mechanisms of CMM are multifactorial and complex, constituting a complicated net of multiple risk factors, ultimately leading to the uncontrolled cancer cell growth [8]. Differentially expressed genes might have a role in the malignancy of CMM, which might serve as therapeutic targets for CMM [4, 9]. Hence, there is a need to have a clear understanding of the underlying molecular mechanisms of CMM progression, which can contribute to diagnosis and therapy.

Recently, studies have identified long non-coding RNAs (lncRNAs) transcribed in the human genome with the advancement of high-throughput sequencing technologies [10, 11]. lncRNAs are more than 200 nucleotides in length and have no or limited protein-coding capacity [12]. New evidence suggests that LncRNAs function differently in various aspects of gene and protein, acting as molecular regulators of a variety of physiological and pathological processes, like organ development, tumorigenesis and tumor progression [13, 14]. Nowadays, increasing researches have reported that a number of lncRNAs might play multiple functions in the initiation and progression of CMM [15, 16]. LINC01296 was found to be aberrantly expressed in several malignancies, including hepatocellular carcinoma, osteosarcoma and oral squamous cell carcinoma, and LINC01296 could also regulate tumorigenesis and progression of cancers [17]. We also observed aberrant expression of LINC01296 in CMM by GEPIA data analysis. However, the functions and mechanisms of lncRNA LINC01296 in the progression of CMM remains unknown. We thus intended to study the underlying molecular mechanism of LINC01296 in the proliferation of CMM.

## MATERIALS AND METHODS

### Bioinformatics analysis

RNA-seq data for CMM and matched normal samples were obtained from the Gene Expression Profiling Interactive Analysis (GEPIA) database for analysis. The expression of lncRNAs was quantified by date through a custom data analysis pipeline that included a series of steps such as quality control, alignment and expression quantification. In addition, to further investigate the underlying molecular mechanisms of LINC01296 in regulating CMM, we used MIRDB (http://www.mirdb.org/), PITAR (https://pictar.mdc-berlin.de/), and BLAST (http://blast.ncbi.nlm.nih.gov/) together to predict three potential conjugated miRNAs of LINC01296.

### Clinical specimens

In total, 30 matched pairs of tumor tissues and adjacent non-cancerous tissues were surgically acquired from CMM patients. All CMM patients had not undergone other treatment related to this disease prior to surgery. All the samples were frozen instantly in liquid nitrogen after surgery and preserved at -80° C, pending subsequent analysis. Clinical data were collected from our institution. This study was approved by our institution review committee.

### Cell culture

The human CMM cell lines (A-375, M21, SK-MEL-2 and A2058) and Human Epidermal Melanocytes, adult cell line (HEMa-LP) were cultured in our laboratory. All cells were cultured following the protocol of the American Type Culture Collection (ATCC, Manassas, VA, USA) recommendations. Besides, all cell lines were cultured in incubator with 5% CO_2_ at 37° C.

### Cell transfection

To construct knockdown or over-expression of LINC01296 in M21 or A2058 cells respectively, sh-LINC01296, oe-LINC01296 and miR-324-3p mimic were utilized. The sequences of MAPK1 was cloned into the plasmid pcDNA3.1 to form pcDNA3.1-MAPK1 was designed. The empty pcDNA3.1 was served as negative control. All products were purchased from GenePharma (Shanghai, China) and transfected into cells using Lipofectamine 3000 (Invitrogen, Carlsbad, CA, USA) based on manufacturer’s protocols. Alterations in LINC01296, miR-324-3p and MAPK1 were assessed by qRT-PCR prior to further analysis.

### RNA isolation and quantitative real-time PCR (qRT-PCR)

Total RNA was extracted from CMM tissues or cell lines by RNA Isolater Total RNA Extraction Reagent (Vazyme, China) on the basis of the instructions. Total RNA (1μg) was reverse transcribed to high-quality cDNA by a PrimeScript RT Master Mix (Vazyme Biotech, Nanjing, China). QRT-PCR was carried out using the SYBR Green Mix (Vazyme Biotech). The expression levels of miRNAs of our research were calculated using the All-in-One™ miRNA qRT-PCR Detection Kit (Vazyme Biotech, Nanjing, China). All qRT-PCR assays were carried out by ABI 7500 system (Applied Biosystems, Foster City, CA, USA). The housekeeping gene glyceraldehyde 3-phosphate dehydrogenase (GAPDH) or SNORD6 (U6 snRNA) were served as reference genes to standardize the expression levels of genes or miRNA. Data analysis was performed using the 2^-ΔΔCt^ method by Applied Biosystems StepOne Plus Real-Time PCR System (Applied Biosystems, USA). QRT-PCR primers are shown below.

LINC01296

Forward: 5’-AGTTCCACCAAGTTTCTTCA-3’,

Reverse: 5’-AGGTTTTGCCAGCATAGAC-3’;

MAPK1

Forward: 5’-AACAGGCTCTGGCCCACCCA-3’,

Reverse: 5’-AGTCCTCTGAGCCCTTGTCCTGA-3’;

GAPDH

Forward: 5’-GCTCTCTGCTCCTCCTGTTC-3’,

Reverse: 5’-ACGACCAAATCCGTTGACTC-3’;

miR-324-3p

Forward: 5’-CGGCGGACTGCCCCAGGTGC-3’,

Reverse: 5’-CGAATTCTAGAGCTCGAGGCAGG-3’;

U6

Forward: 5’-CTCGCTTCGGCAGCACA-3’,

Reverse: 5’-AACGCTTCACGAATTTGCGT-3’.

### Cell proliferation assay

Cell proliferation was examined by the Cell Counting Kit-8 (CCK-8; Dojindo Laboratories, Kumamoto, Japan). M21 and A2058 cells were seeded in 96-well plates at a density of 3000 cells/well, and then incubated for 0, 24, 48, and 72 h. Following that, 20 μL CCK-8 was added to each well, incubated for 2 h. The absorbance was determined at the wavelength of 450 nm.

### Colony formation assay

Cells were transfected in a six-well plate at a density of 200 cells/well and cultured for almost 2 weeks at 37° C and 5% CO_2_ to complete the colony formation assay. Colonies were fixed with 4% paraformaldehyde, stained with 0.5% crystal violet and subsequently counted using Image J.

### Ethynyl-2’-deoxyuridine (EDU) assay

The proliferative ability of CMM cell lines was tested by an EDU kit (Abcam, Cambridge, MA, USA) following the user manual. Cells were grown on coverslips and cultured with EDU for 2 hours during DNA synthesis, then stained with anti-EDU antibody after treatment. Images were acquired under a microscope with an Olympus camera.

### Dual luciferase reporter assay

LINC01296 wild-type (WT) containing miR-324-3p binding sites on the LINC01296 promoter region and LINC01296 mutant type (MUT), were ligated into pGLO vectors respectively, named pGLO-LINC01296-WT and pGLO-LINC01296-MUT. Either pGLO-LINC01296-WT or pGLO-LINC01296-MUT was co-transfected with miR-324-3p mimic or NC mimic into HEK-293FT cells. After 24h, the cells were collected and lysed. Besides, the same conduction did on MAPK1. Firefly and Renilla luciferase activities were measured with Dual-Luciferase Reporter Assay System (Promega, Madison, WI, USA).

### Tumorigenicity assays in nude mice

The indicated stable M21 cell lines (2×10^6^) were subcutaneously injected into the right flank of BALB/c (nu/nu) 4- to 6-week-old female nude mice (N=5). Tumor size was measured once per 4 days and mice were sacrificed to analyze the tumor burden after 4 weeks and tumor volume was calculated using the formula: Tumor volume = 1/2(length × width^2^). All procedures of animal experiments were performed in accordance with the Animal Ethics Committee of Nanjing Medical University.

### Immunohistochemical staining

Hematoxylin and eosin (H&E) staining was utilized to select representative areas. Tissue samples embedded in paraffin were stained to identify and measure the expression levels of MAPK1 and Ki-67. The tumor tissues were detected with primary monoclonal probes for MAPK1 and Ki-67 overnight at 4° C. After incubation with a suitable second antibody, the tissue microarrays were treated with diaminobenzidine and counterstained with hematoxylin. The sections were visualized under a microscope (400×) (Olympus, Japan). The results were graded according to the percentage of positive cells.

### Western blotting

Total proteins from M21 and A2058 cells were obtained utilizing RIPA buffer containing protease inhibitors and phosphatase inhibitors. The lysates were centrifuged on ice for 30 min and then at 12,000 rpm for 15 min at 4° C. Protein concentrations were determined using a BCA kit (Beyotime Biotechnology, Beijing, China). The isolated proteins were transferred to PVDF membrane by SDS-PAGE for separation. After that, the membranes were blocked with 5% skim milk and incubated with anti-MAPK1 primary antibody at 4° C overnight. Later, the membranes were incubated with the corresponding secondary antibodies at room temperature for 1 h. GAPDH expression levels served as a loading control. Protein bands were visualized using a chemiluminescent reagent (ECL) kit (Beyotime Biotechnology) following the product instructions. All antibodies were obtained from Servicebio (Wuhan, China).

### Statistical analysis

The data were presented as mean ± SD and analyzed in GraphPad Prism 7.0 (GraphPad Software, La Jolla, CA, USA). One-way ANOVA or two-tailed Student’s t-test was conducted for P-value analysis, depending on the situation. In present paper, data from the control group were selected as a normalized control on the y-axis. Unless otherwise stated, every experiment was performed at least in triplicate. *P*<0.05 was regarded as statistically significant. We followed the methods of Kai Zhu et al. 2020 [18].

### Consent for publication

The patient gave an informed consent for this publication.

## RESULTS

### Identification of LINC01296 as an up-regulated lncRNA in CMM

**Figure 1:**
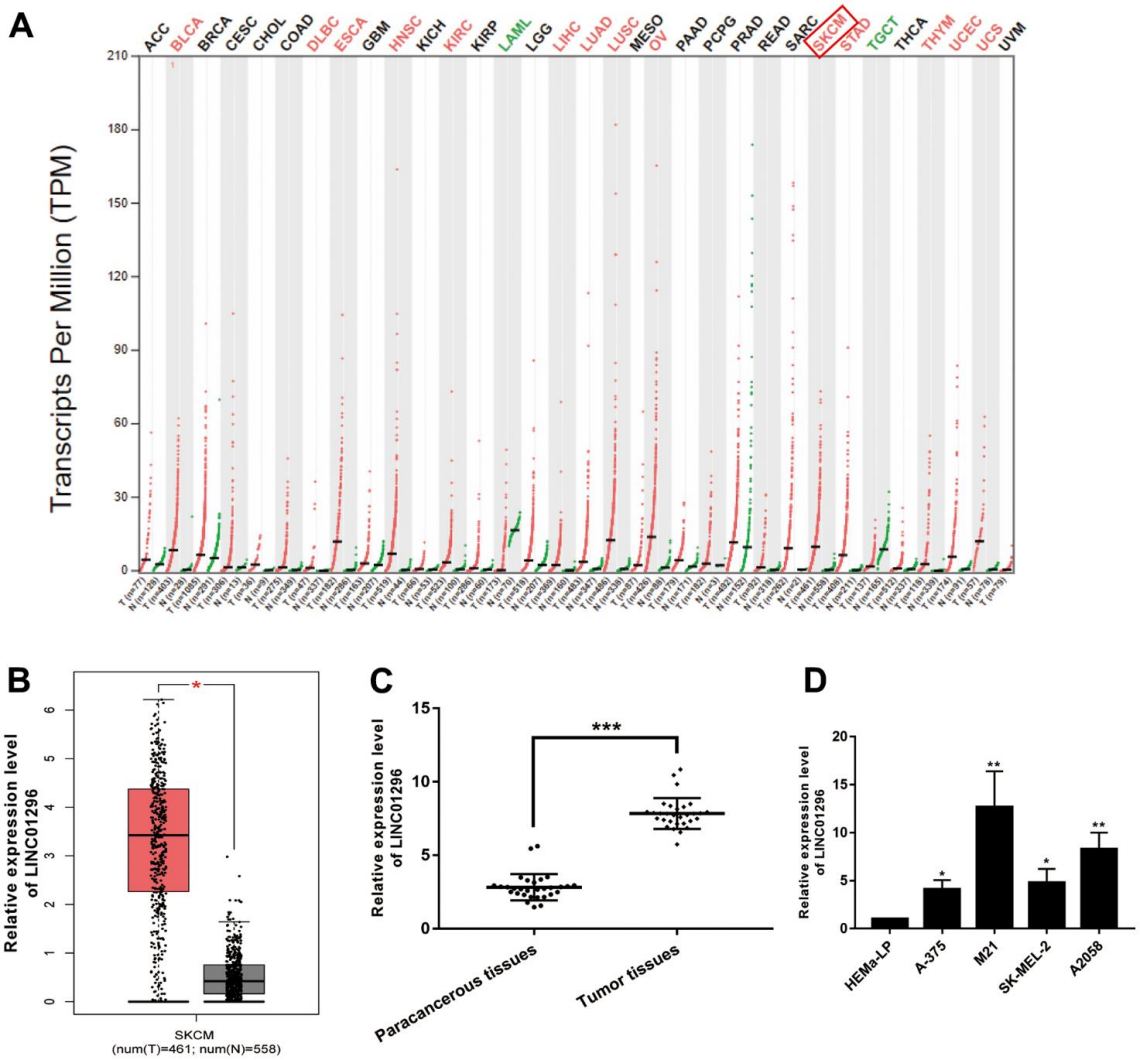
**Identification of LINC01296 as an up-regulated lncRNA in CMM.** (**A**) GEPIA database results about the up-expression of LINC01296 in CMM tissues. (**B**) LINC01296 expression in CMM tissues and adjacent non-cancerous tissues from GEPIA database. (**C**) qRT-PCR analysis of the expression level of LINC01296 in 30 paired CMM tissues and the adjacent non-cancerous tissues. (**D**) LINC01296 expression level in CMM cell lines and human epidermal melanocytes, adult cell line (HEMa-LP) were detected by qRT-PCR analysis. All of data were analyzed from three independent experiments. * *P* < 0.05; ** *P* < 0.01,*** *P* <0.001 vs. control group.

According to the analysis of the Gene Expression Profile Interactive Analysis (GEPIA) database, a high expression of LINC01296 was observed in a variety of cancers, including CMM (Figure 1A). Moreover, LINC01296 expression was markedly upregulated in cutaneous melanoma (SKCM) (Figure 1B, P<0.05), which was in accordance with the data of NCBI (https://www.ncbi.nlm.nih.gov/). We collected 30 pairs of CMM tumor tissues and adjacent non-cancerous tissues to further investigated the function of LINC01296 in CMM tumorigenesis, and we examined the expression level of LINC01296. Based on the results of qRT-PCR, it was shown in the Figure 1C, CMM tissues exhibited higher level of LINC01296, compared to adjacent non-cancerous tissues (*P*<0.001). Moreover, compared with human Epidermal Melanocytes, adult cell line HEMa-LP, LINC01296 was highly expressed in CMM cell lines (A-375, M21, SK-MEL-2 and A2058) to varying degrees, among which M21 cell lines exhibited highest expression level of LINC01296, whereas A2058 cell lines in the second (Figure 1D). In summary, our results showed that LINC01296 was markedly overrepresented in CMM tissues and cell lines, and it may serve as an oncogene in CMM.

### LINC01296 promoted the proliferation abilities of CMM cells

**Figure 2:**
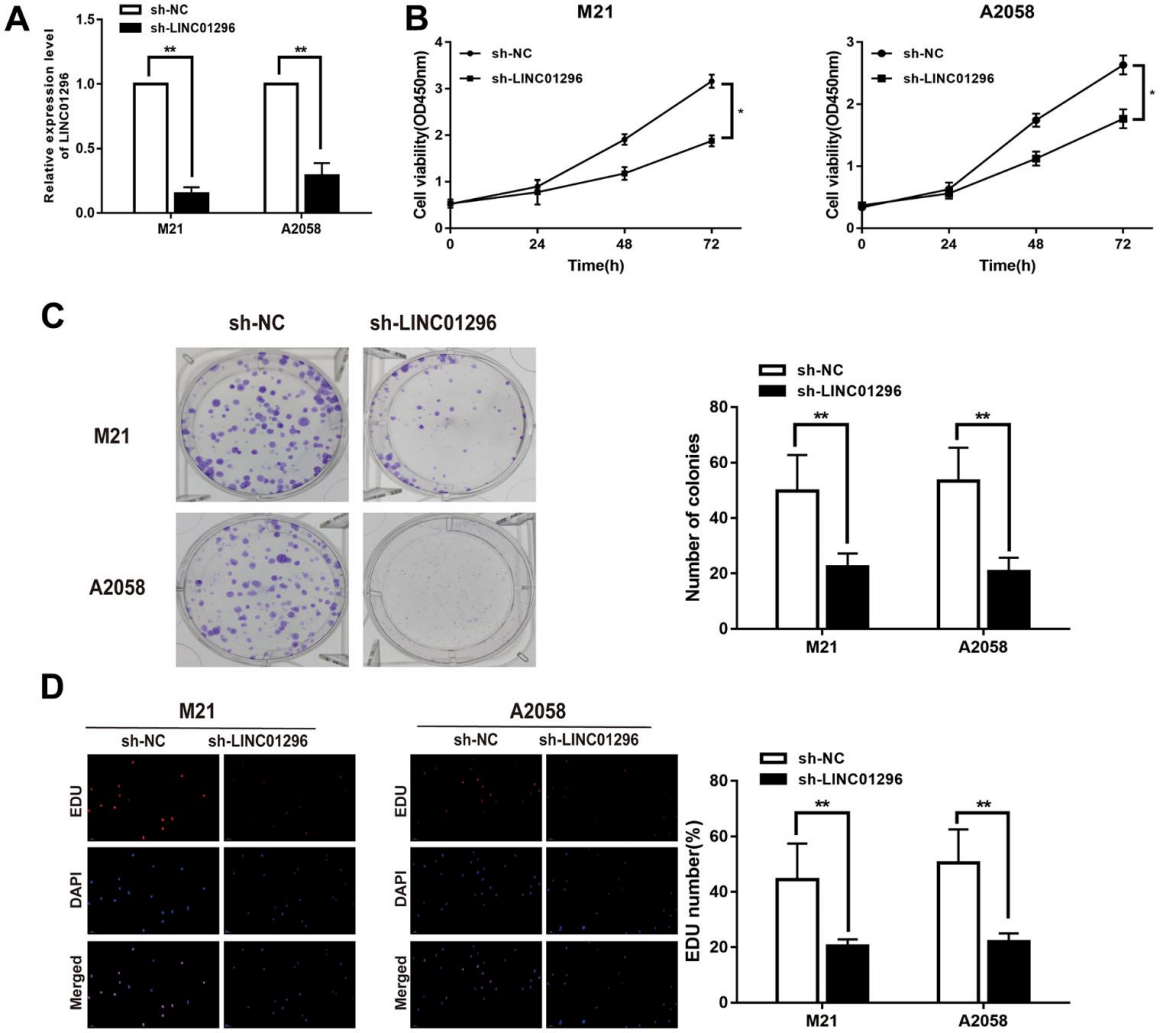
**Knock-down of LINC01296 decreased proliferation abilities of CMM cells.** (**A**) The relative expression of LINC01296 determined by qRT-PCR analysis following the treatment of knocking down LINC01296 (sh-LINC01296) in M21 and A2058 cells. (**B**) Cell proliferation was examined by CCK-8 assays in sh-LINC01296 group at the indicated time points in M21 and A2058 cells. The sh-NC was as control. (**C**) Cell proliferation was determined by colony-formation assay of impacts of knocking down LINC01296 in M21 and A2058 cells. (**D**) EDU assay was used to assess cell proliferation of knocking down LINC01296 in M21 and A2058 cells. All of data were analyzed from three independent experiments. * *P* < 0.05; ** *P* < 0.01.

**Figure 3:**
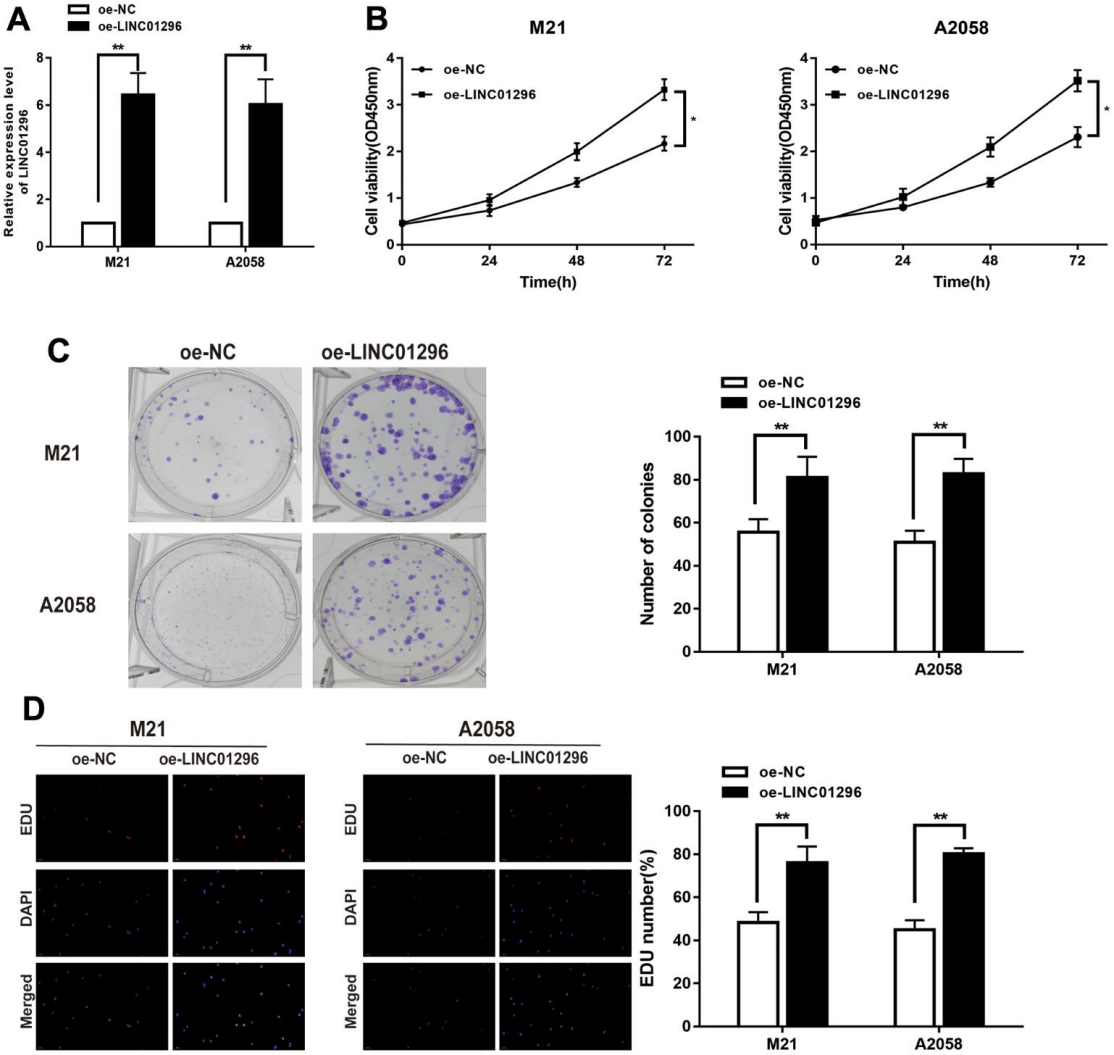
**Over-expression of LINC01296 increased proliferation abilities of CMM cells.** (**A**) The relative expression of LINC01296 determined by qRT-PCR analysis following the treatment of over expressing LINC01296 (oe-LINC01296) in M21 and A2058 cells. (**B**) Cell proliferation was examined by CCK-8 assays in oe-LINC01296 group at the indicated time points in M21 and A2058 cells. The oe-NC was as control. (**C**) Cell proliferation was determined by colony-formation assay of impacts of over expressing LINC01296 in M21 and A2058 cells. (**D**) EDU assay was used to assess cell proliferation of over expressing LINC01296 in M21 and A2058 cells. All of data were analyzed from three independent experiments. * *P* < 0.05; ** *P* < 0.01.

To further analysis, the knock-down model of LINC01296 with sh-LINC01296 and over-expression model of LINC01296 with oe-LINC01296 were established, in M21 and A2058 cells, respectively (Figures 2A, 3A, *P*<0.01). A further study regarding the biological effect of LINC01296 on the proliferative capacity of CMM, CCK-8 experiments showed that knockdown of LINC01296 suppressed the proliferation capacity of M21 and A2058 cells (Figure 2B, P<0.05). By contrast, overexpression of LINC01296 enhanced the proliferation capacity of CMM cell lines (Figure 3B, *P*<0.05). Similarly, cell proliferation was examined by colony formation assay, showing that cell colonies in the sh-LINC01296 group was much smaller than in the sh-NC group (Figure 2C, *P*<0.01), whereas cell colonies were markedly larger in the oe-LINC01296 group compared to the oe-NC group (Figure 3C, *P*<0.01). This fact highlights that LINC01296 enhanced the expansion capacity of CMM cell lines. Furthermore, the regulation of LINC01296 had a significant effect on proliferation rate of CMM cells, which was measured using EDU assay. Our findings revealed that the proliferation of M21 cells transfected with sh-LINC01296 was diminished (Figure 2D, *P*<0.01), whereas enhanced in M21 and A2058 cells transfected with oe-LINC01296 (Figure 3D, *P*<0.01). Taken together, these results suggested that LINC01296 possessed tumor-inducing activity in the progression of CMM.

### knock-down of LINC01296 contributed to suppress tumorigenesis *in vivo*

**Figure 4:**
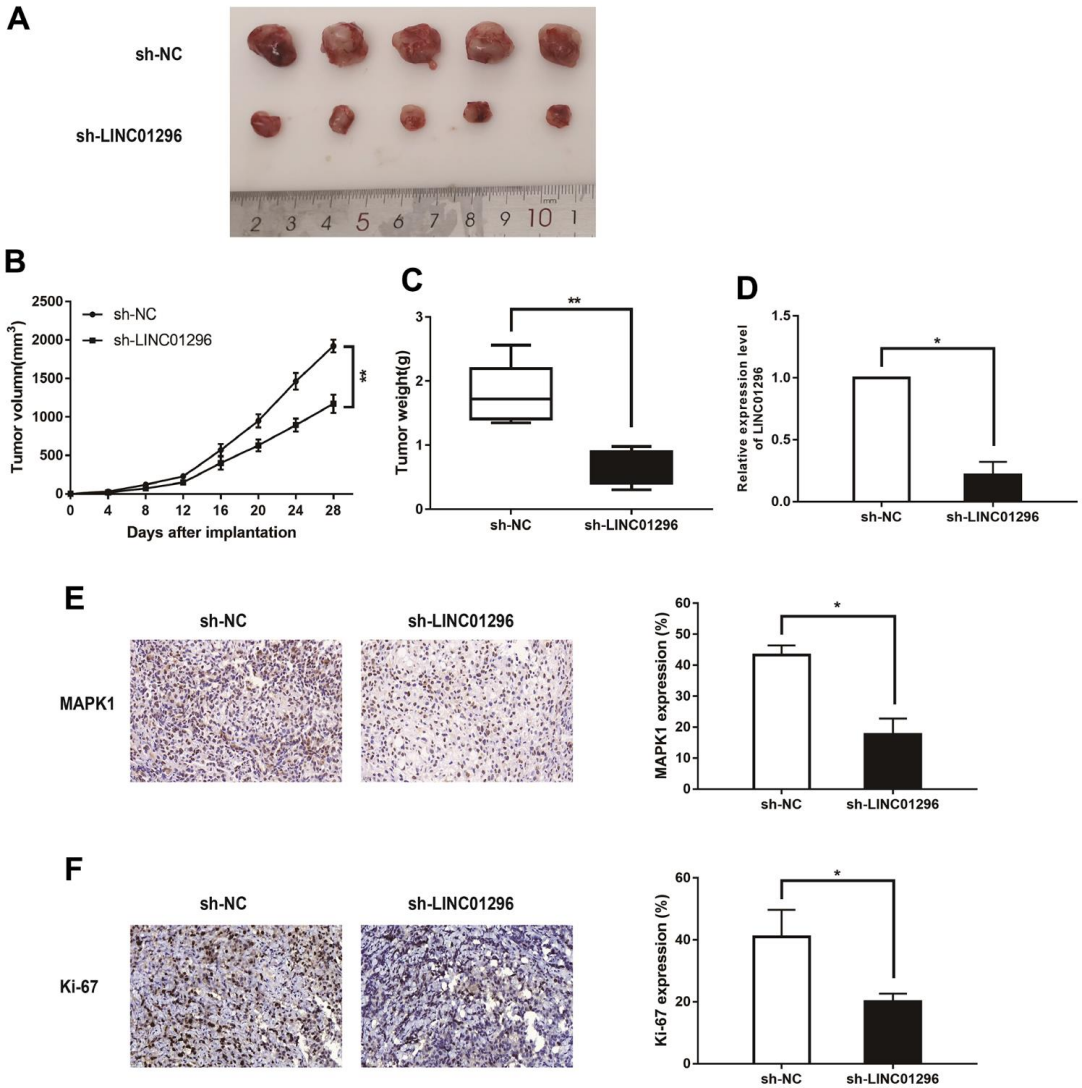
**Decreasing LINC01296 contributed to suppress tumorigenesis *in vivo*.** (**A**) Representative images of the xenograft tumors in subcutaneous xenograft mouse model injected with M21 cells transfected with sh-NC and sh-LINC01296. (**B**) Tumor volume of the xenograft in each group. (**C**) Tumor weight of the xenograft in each group. (**D**) The relative expression level of LINC01296 determined by qRT-PCR analysis following transfecting with sh-NC and sh-LINC01296. (**E**) The tumor sections from different transfected groups of xenograft mouse models were subjected to immunohistochemistry staining using antibodies against MAPK1 (400×). (**F**) The tumor sections from different transfected groups of xenograft mouse models were subjected to immunohistochemistry staining using antibodies against ki-67 (400×) All of data were analyzed from three independent experiments. * *P* < 0.05; ** *P* < 0.01.

To conduct for the purpose of exploring the functions of LINC01296 in tumor growth *in vivo*, Figure 4A showed the representative images of the xenograft tumors in subcutaneous xenograft mouse model injected with M21 cells transfected with sh-NC and sh-LINC01296 (N=5 nude mice per group). The knockdown of LINC01296 caused less tumor formation and significantly decreased tumor size compared with sh-NC group (Figure 4B, 4C, *P*<0.01). In addition, we also found that the expression level of LINC01296 in sh-LINC01296-xenografted tumors was significantly lower than that in sh-NC-xenografted tumors by qRT-PCR assay (Figure 4D, *P*<0.05). In accordance with our previous data, immunochemistry analysis confirmed that MAPK1 expression level in sh-LINC01296-xenografted tumors decreased compared to sh-NC-xenografted tumors (Figure 4E, *P*<0.05). Moreover, tumor section of sh-LINC01296 group presented lower positive rate of Ki-67 than that of sh-NC group (Figure 4F, *P*<0.05). Our work emphasized that inhibition of LINC01296 inhibited CMM tumorigenesis and tumor development *in vivo*.

### LINC01296 interacted with miR-324-3p and repressed its expression in CMM

**Figure 5:**
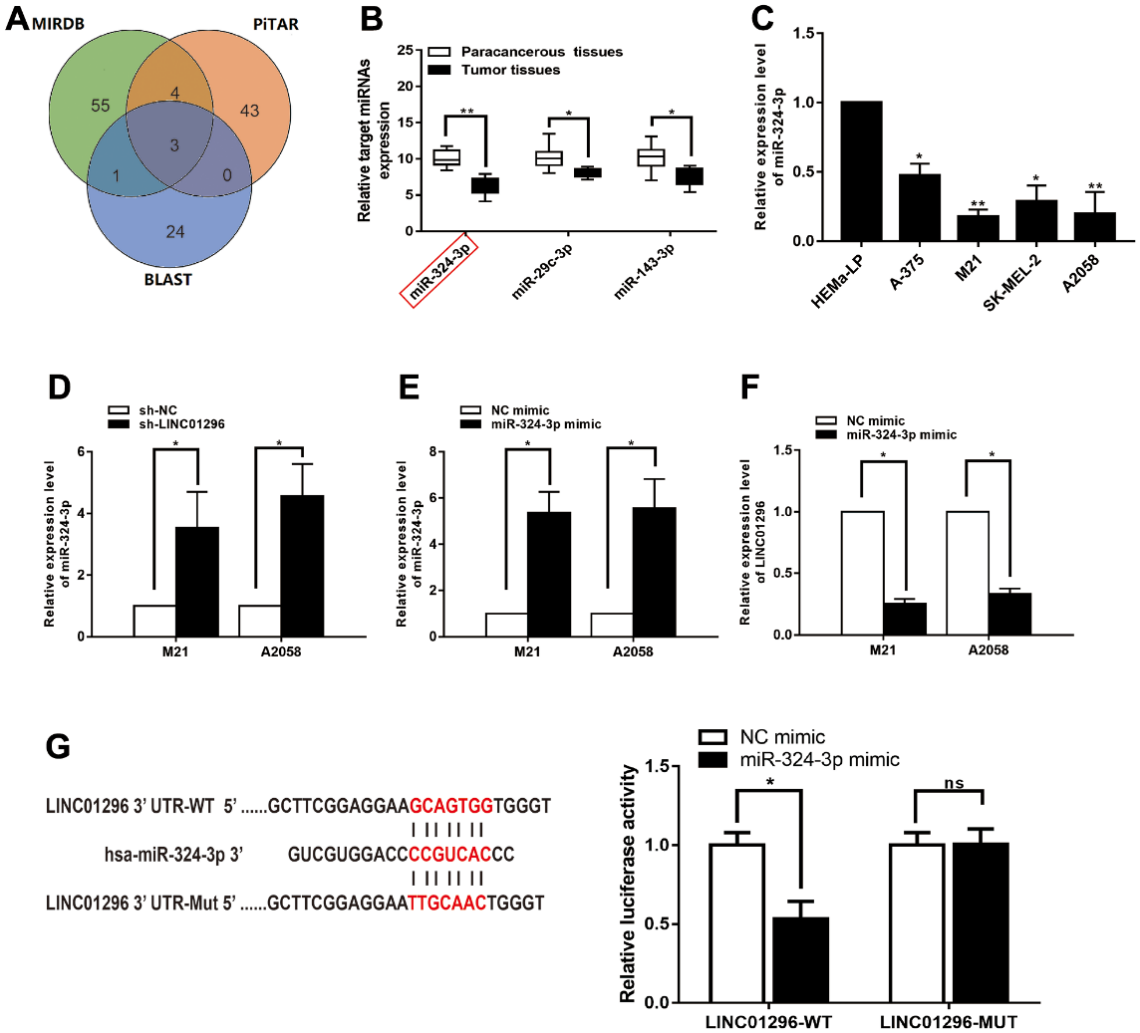
**LINC01296 interacted with miR-324-3p and repressed its expression in CMM.** (**A**) A schematic diagram used to search the target miRNAs of LINC01296 in three databases. (**B**) qRT-PCR assay confirmed the relative expression of three candidate miRNAs of LINC01296 in 30 paired CMM tissues compared with adjacent non-cancerous tissues. (**C**) miR-324-3p expression level in CMM cell lines and human epidermal melanocytes, adult cell line (HEMa-LP) were detected by qRT-PCR analysis. (**D**) Schematic illustration of the predicted binding sites between LINC01296 and miR-324-3p, and mutation of potential miR-324-3p binding sequence in LINC01296. Relative luciferase activities of wild type (WT) and mutated (MUT) LINC01296 reporter plasmid in human embryonic kidney (HEK) 293T cells co-transfected with miR-324-3p mimic. (**E**) The relative expression of miR-324-3p in M21 and A2058 cells transfected with knocking down LINC01296 by qRT-PCR analysis. (**F**) The relative expression of miR-324-3p in M21 and A2058 cells transfected with miR-136-5p mimic by qRT-PCR analysis. (**G**) The relative expression of LINC01296 in M21 and A2058 cells transfected with miR-136-5p mimic by qRT-PCR analysis. All of data were analyzed from three independent experiments. *P < 0.05, ** *P* < 0.01, ^ns^ *P*>0.05.

Bioinformatics analysis (BA) showed that three potential conjugated miRNAs of LINC01296 were predicted, based on MIRDB, PITAR, and BLAST databases (Figure 5A). Among the three related miRNAs, miR-324-3p was significantly decreased verified in 30 CMM tissues compared with pair-normal tissues by qRT-PCR analysis (Figure 5B, *P*<0.01). Furthermore, qRT-PCR assay was utilized to validated miR-324-3p expression in CMM cell lines and found it was significantly decreased in M21 and A2058 cells (Figure 5C). We further investigated whether miR-324-3p was truly a ceRNA for LINC01296. In addition, we found that miR-324-3p expression was significantly upregulated in the knockdown model of LINC01296 (Figure 5D, P<0.05). Then, overexpression efficiency of miR-324-3p mimics in M21 and A2058 cells was tested by qRT-PCR analysis (Figure 5E, *P*<0.05), and miR-324-3p mimics could lead to downregulation of LINC01296 expression (Figure 5F, *P*<0.05). We used a dual luciferase reporter gene assay to evaluate whether LINC01296 acts on miR-324-3p. It can be seen in Figure 5G, the luciferase activity of cells transfected with miR-324-3p mimics was significantly declined than in the NC mimic group (*P* < 0.05), while no remarkable variation was observed in luciferase activity in cells transfected with miR-324-3p mimic in LINC01296-MUT group (*P*<0.05).

### MAPK1 was a direct target of miR-324-3p in the progression of CMM

**Figure 6:**
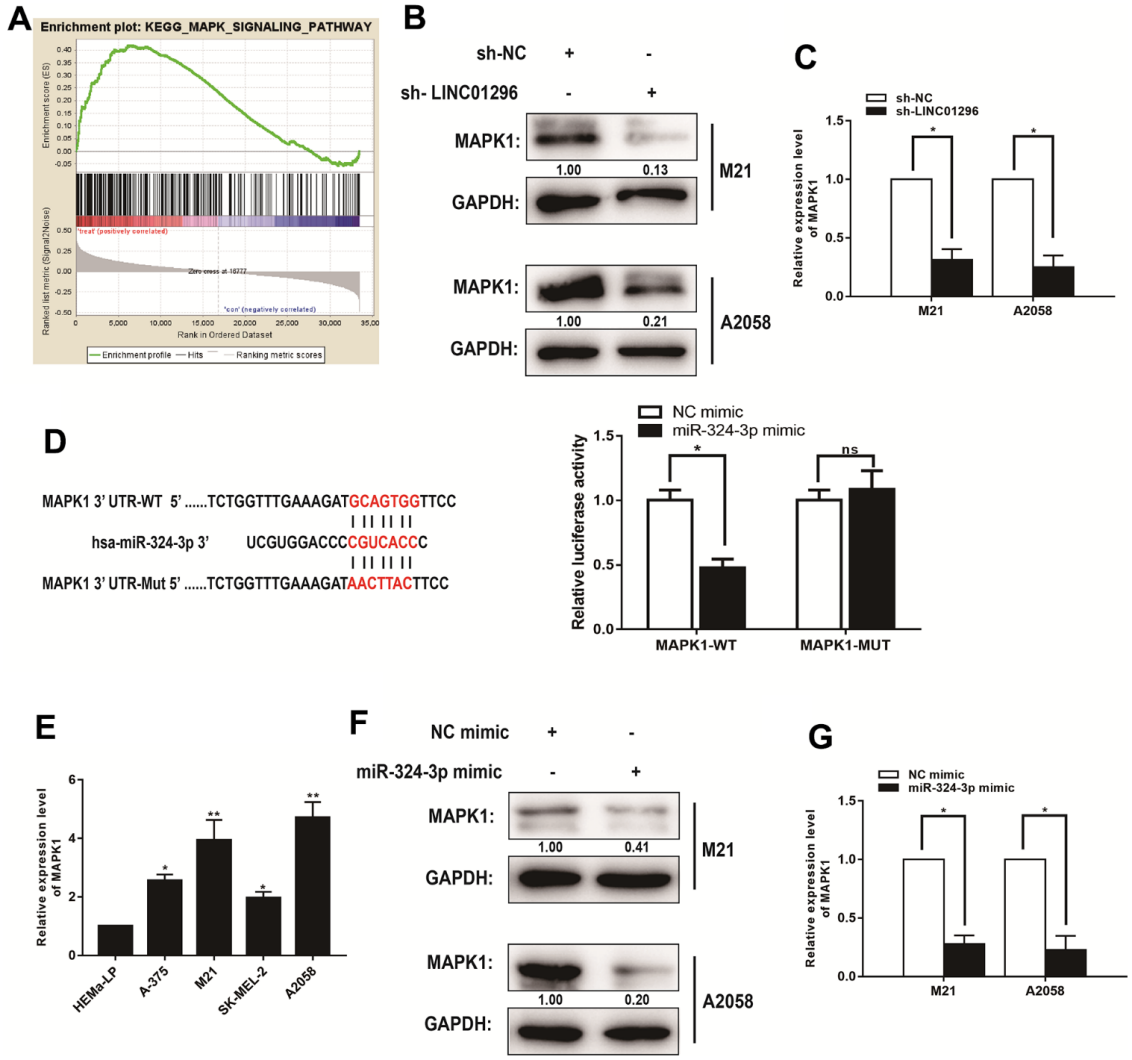
**MAPK1 was a direct target of miR-324-3p in CMM progression.** (**A**) Kyoto Encyclopedia of Genes and Genomes analysis revealed that the MAPK signaling pathway was significantly altered in CMM. (**B**) The effect of LINC01296 on MAPK1 expression was analyzed by Western blotting with the indicated antibodies and samples from the M21 and A2058 cells transfected with sh-LINC01296 or sh-NC. (**C**) The relative expression of MAPK1 in M21 and A2058 cells transfected with knocking down LINC01296 by qRT-PCR analysis. (**D**) Schematic illustration of the predicted binding sites between miR-324-3p and MAPK1, and mutation of potential miR-324-3p binding sequence in MAPK1. Relative luciferase activities of wild type (WT) and mutated (MUT) MAPK1 reporter plasmid in human embryonic kidney (HEK) 293T cells co-transfected with miR-324-3p mimic. (**E**) MAPK1 expression level in CMM cell lines and human epidermal melanocytes, adult cell line (HEMa-LP) were detected by qRT-PCR analysis. (**F**) The relative expression of MAPK1 in M21 and A2058 cells transfected with miR-136-5p mimic by Western Blotting analysis. (**G**) The relative expression of MAPK1 in M21 and A2058 cells transfected with miR-136-5p mimic by qRT-PCR analysis. All of data were analyzed from three independent experiments. *P < 0.05, ** *P* < 0.01, ^ns^ *P*>0.05.

The lncRNAs have been shown to function critically in regulating the progression of CMM through MAPK signaling pathway. To determine the potential downstream genes that contributes to the effects of LINC01296 on CMM cells, we obtained mRNAs for potentially related genes of LINC01296 by BA. Moreover, we explored the signaling pathways that potential related mRNAs involved in by using Kyoto Encyclopedia of Genes and Genomes analysis. It is noteworthy that the MAPK signaling pathway was significantly altered in CMM, which was in accordance with the findings *in vitro* (Figure 6A). To test the effect of LINC01296 knock-down on MAPK signaling pathway, the results by Western blotting indicated that the knock-down of LINC01296 could blunt MAPK signaling pathway in both M21 and A2058 cells. Compared with sh-NC group, we exhibited the decreasing expression level of MAPK1 in both M21 and A2058 cells transfected with sh-LINC01296 group (Figure 6B). And as shown in qRT-PCR assay, the knock-down of LINC01296 resulted in the down-regulation of MAPK1 expression (Figure 6C, *P*<0.05). Finally, we performed dual-luciferase reporter gene assay, and the results found that the over-expression of miR-324-3p decreased the luciferase activity driven by MAPK1-WT in HEK293FT cells, but did not change the activity of MAPK1-MUT, which suggested that MAPK1 was a direct target of miR-324-3p (Figure 6D, *P*<0.05). In addition, further verification of MAPK1 was highly-expressed in M21 and A2058 cells by qRT-PCR (Figure 6E). Then, to validate the correlation between miR-324-3p and MAPK1, miR-324-3p mimic was established in M21 and A2058 cells, and the results by Western Blotting and qRT-PCR showed the decreasing expression level of MAPK1 in M21 and A2058 cells transfected with miR-324-3p mimic, which revealed that miR-324-3p could regulate MAPK1 level (Figure 6F, 6G, *P*<0.05).

### LINC01296 promoted tumorigenesis in CMM via modulating MAPK1

**Figure 7:**
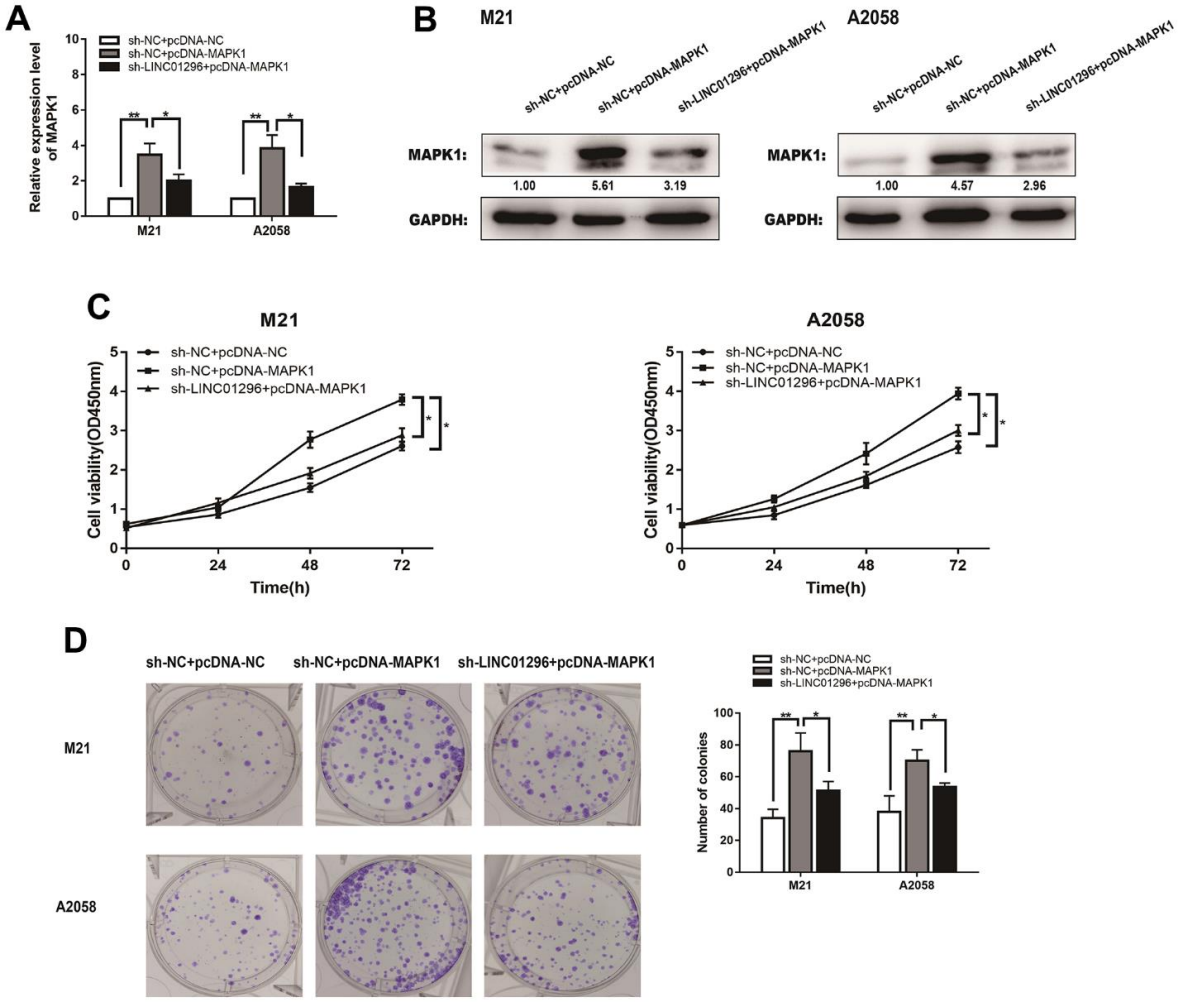
**LINC01296 enhanced the proliferation abilities of CMM cells by regulating MAPK1.** (**A**) qRT-PCR was conducted to verify the relative expression of MAPK1 in M21 and A2058 cells transfected with sh-NC+pcDNA-NC, sh-NC+pcDNA-MAPK1, sh-LINC01296+pcDNA-MAPK1. (**B**) The expression of MAPK1 was analyzed by Western blotting with the indicated antibodies and samples from the M21 and A2058 cells in different transfected groups. (**C**) CCK-8 assay of M21 and A2058 cells in different transfected groups. (**D**) Cell proliferation was determined by colony-formation assay of different transfected groups in M21 and A2058 cells. All of data were analyzed from three independent experiments. * *P* < 0.05; ** *P* < 0.01.

The expression level of MAPK1 of M21 and A2058 cells from different transfection groups were measured by qRT-PCR (Figure7A). MAPK1 expression levels were lower in sh-LINC01296+pcDNA3.1 group of CMM cell lines than that in sh-NC+pcDNA3.1 group, whereas they were obviously higher in sh-LINC01296+pcDNA3.1-MAPK1 group of M21 and A2058 cells. Western blotting showed a same phenomenon (Figure 7B). Functional assays, like CCK8 assays and cloning assays, were also carried out in different transfection groups of M21 and A2058 cells (Figure 7C, 7D). Based on the experimental results, it can be seen that MAPK1 expression has the ability to reverse the opposite effect of LINC01296 in CMM cells, suggesting that upregulation of LINC01296 can induce tumorigenesis in CMM by upregulating the miR-324-3p/MAPK1 axis.

## DISCUSSION

Malignant melanoma frequently occurs in the skin and has become one of the common malignant tumors of the skin [2, 3]. Although the incidence rate of CMM is lower than that of cutaneous basal cell carcinoma and squamous cell carcinoma of the skin, its malignant degree is significantly higher than the former [1–3]. The incidence rate of CMM increases year by year, with a younger disease onset [2]. CMM is commonly progressed from benign sputum; however, in the later stage, it is characterized as rapid development, high invasion, frequent metastasis and recurrence [1, 6]. The patients with CMM are often diagnosed as advanced stage due to the inadequate attention, which is explained as one of the reasons for its poor prognosis and high mortality [19]. So due to the unsatisfaction of relatively poor diagnosis and prognosis of CMM, numerous studies on association between the related factors and the treatment of CMM have been receiving increasing attention [20, 21]. Therefore, searching for these novel treatments are of great significance for CMM. There is growing evidence that lncRNAs play a broad regulatory role as active biomolecules in the carcinogenesis and progression of CMM [14, 15, 20]. Depending on the location, lncRNAs can achieve their functions by being implicated in the production and progression of target genes on various levels [14, 16]. In this study, we found that lncRNA LINC01296 is highly overexpressed in the progression of CMM. However, the underlying functions and molecular mechanisms of LINC01296 in CMM progression remain unknown.

Firstly, our results showed that the expression level of LINC01296 was significantly upregulated in CMM tissues relative to adjacent non-cancerous tissues in the GEPIA database, which is consistent with the results of clinical CMM specimens. In addition, we analyzed the tumorigenic effect of LINC01296 in CMM cells by *in vitro* cell function assays, which showed that knockdown of LINC01296 in M21 and A2058 cells significantly suppressed tumor proliferation. On the contrary, overexpression of LINC01296 in CMM cell lines potently increased the proliferation of CMM cells. Thus, our study reveals that LINC01296 is an oncogene of CMM and may be a promising diagnostic and therapeutic indicator of CMM.

In the ensuing research, we intended to reveal the underlying mechanisms by which LINC01296 influences tumorigenesis and progression in CMM. LncRNAs may act as decoys for miRNAs and their target genes, or guiding RNA binding protein of transcription factors or epigenetic regulation [22, 23]. To extend our investigation into possible conjugative miRNAs for LINC01296, we found that miR-324-3p was in the intersection of MiRDB, PITA and BLAST databases. The BA results revealed that miR-324-3p bound to LINC01296, and this was confirmed by qRT-PCR and dual luciferase reporter assays. Although miR-324-3p is a key regulator of human cancer progression, there are no researches on the role and molecular regulatory mechanisms of miR-324-3p in CMM. Besides, interaction between the expression levels of LINC01296 and miR-324-3p was validated in CMM cell lines, indicating that miR-324-3p functions as ceRNA for LINC01296.

MAPK signaling pathway is a well-known pathway in tumor proliferation. MAPK1 encodes extracellular signal-regulated kinases (ERKs), which belong to the MAP kinase family. As an integration site for multiple biochemical signals, it is involved in numerous cellular processes including proliferation, differentiation and development [24, 25]. the MAPK signaling pathway participates in early and late development, including organogenesis and morphological determination [26, 27]. Our analysis revealed that miR-324-3p can interact with MAPK1, which in turn regulates MAPK1 expression. Besides, we observed that knockdown of LINC01296 effectively inhibited MAPK1 expression and impaired cell growth. Furthermore, MAPK1 has been considered to be active in multiple signaling pathways in human tumors. The collected clinical samples confirmed that upregulated LINC01296 effectively increased MAPK1 expression levels. Taking a step further, *in vitro* experiments validated that protein MAPK1 associated with MAPK signaling pathway were up-regulated in the over-expression of LINC01296. Therefore, we concluded that MAPK1 acted as a downstream gene of LINC01296 and miR-324-3p bind to MAPK1, which LINC01296 could regulate miR-324-3p and enhance MAPK signaling pathway, then contribute to tumorigenesis of CMM.

With the aim of proving the axis that LINC01296 boosted MAPK1 expression in CMM through sponging effect on miR-324-3p, a rescue experiment was then developed to uncover the role of LINC01296/miR-324-3p/MAPK1 axis in CMM. The experiments suggested that upregulation of MAPK1 expression reverted the tumor-promoting role of LINC01296 in CMM. These findings offered evidence that LINC01296 could be a promising bio-diagnostic marker and a key molecular target for tumor progression in CMM.

## CONCLUSIONS

To sum up, this paper shows that LINC01296 can be an oncogene of CMM by disrupting the interaction between miR-324-3p and MAPK1. Mechanistically, our work showed that LINC01296 directly sponged miR-324-3p, and promoted MAPK1 expression, to exacerbated the proliferation of CMM. Together, the characterization of LINC01296/miR-324-3p/MAPK1 axis might bring us novel perspectives for diagnostic and prevention progress of CMM.
